# Association of monoaminergic gene polymorphisms in chronic inflammatory pulmonary disease patients with successful smoking cessation

**DOI:** 10.1186/s12890-024-03219-y

**Published:** 2024-08-26

**Authors:** Angela Mikaczo, Csaba Papp, Tamas Erdei, Aniko Posa, Gabor Zahuczky, Csaba Varga, Janos Szabo, Rudolf Gesztelyi, Maria Szilasi, Judit Zsuga

**Affiliations:** 1 Department of Pulmonology, Faculty of Medicine, University of Debrecen, Nagyerdei krt. 98, Debrecen, H-4032 Hungary; 2 Doctoral School of Pharmaceutical Sciences, University of Debrecen, Debrecen, H-4032 Hungary; 3 Department of Psychiatry, Faculty of Medicine, University of Debrecen, Nagyerdei krt. 98, Debrecen, H- 4032 Hungary; 4 Department of Pharmacology and Pharmacotherapy, Faculty of Medicine, University of Debrecen, Nagyerdei krt. 98, Debrecen, H-4032 Hungary; 5 Department of Oral Biology and Experimental Dental Research, Faculty of Dentistry, University of Szeged, Tisza Lajos krt. 64, Szeged, H-6720 Hungary; 6 Department of Physiology, Anatomy and Neuroscience, Faculty of Science and Informatics, University of Szeged, Közép fasor 52, Szeged, H-6726 Hungary

**Keywords:** Smoking cessation, SNP, rs2235186, Rs4680 G/A COMT polymorphism, Tonic dopamine, Smoking cues

## Abstract

**Background:**

Albeit smoking cessation has unequivocal health benefits, attempts to quit are not unanimous, even in patient populations at high risk for smoking-related diseases cessation. Allelic variations of enzymes involved in dopamine metabolism are being considered as candidates for nicotine addiction. We set out to assess whether rs4680 G/A and rs2235186 G/A polymorphisms of COMT and MAO-A, respectively are associated with the ability to quit smoking in chronic inflammatory pulmonary disease patients.

**Methods:**

Patients managed for chronic inflammatory pulmonary disease by the Department of Pulmonology (University of Debrecen, Hungary), with a current or past smoking habit were included in the current analysis. The study was designed in line with the STROBE statement for cross-sectional studies and was approved by the National Center for Public Health, Hungary. Genomic DNA was extracted from peripheral blood specimens. SNPs were genotyped using TaqMan SNP genotyping assays.

**Results:**

rs4680 COMT polymorphism showed significant effect for successful smoking cessation in patients with pulmonary disease. Accordingly, A/A subjects had lower odds for successful smoking cessation (odds ratio 0.37; 95% confidence interval 0.20–0.69, *p* = 0.002 (additive model). On the other hand, patients homozygous for the minor allele (A) at rs2235186 of MAO-A showed a non-significant trend toward increased odds for successful smoking cessation.

**Conclusions:**

The presence of the minor allele for rs4680 COMT was shown to decrease the odds for successful smoking cessation, a finding that may be interpreted in view of the altered balance between tonic and phasic dopamine release.

**Supplementary Information:**

The online version contains supplementary material available at 10.1186/s12890-024-03219-y.

## Introduction

Globally, smoking (i.e. combustible tobacco use) is one of the most significant preventable causes of premature death. Tobacco-attributable diseases account for over 25% of all years lost due to disability of any cause; for adults aged between 45 and 79 years. Tobacco-related diseases include cardiovascular diseases (e.g. ischemic heart disease, and stroke), cancer (tracheal, bronchial, lung, lip and oral cavity cancer) and chronic inflammatory pulmonary diseases (e.g. bronchial asthma (BA) and chronic obstructive pulmonary disease (COPD)). Of tobacco-related diseases, the latter amount to the second highest number of smoking-attributable deaths, reaching over 1.8 million deaths in total – with 1.7 and 0.1 million deaths attributed to COPD and BA, respectively [1].

Albeit smoking cessation has unequivocal health benefits, attempts to quit are not unanimous, and not without failures. There is a gap between those who wish to stop smoking, those who actually try quitting and those who quit. According to the Tobacco Atlas, approximately 70%, 54.1% and 7.5% of smokers intended to quit smoking, tried quitting, or quit, respectively in 2018, in the United States. [1, 2] This low success rate is in part due to the addictive nature of nicotine. Accordingly, several sociodemographic factors have been identified in relation to smoking, including low level of education, unfavorable socio-economic status, social environmental factors (e.g. smokers in the family and among peers), and starting smoking at an early age [3]. Furthermore, heritability for regular tobacco use and nicotine dependence was estimated to reach 80% and 60%, respectively [4], and genetic factors are responsible for approximately 50% of the variance in cessation success [5]. Failure to quit smoking has been associated with polymorphisms of nicotinic acetylcholine receptors. A recent systemic review summarized the evidence regarding single nucleotide polymorphisms (SNPs) of the nicotinic acetylcholine receptor genes and smoking cessation. Minor alleles of rs16969968 cholinergic receptor nicotinic alpha 5 subunit (CHRNA5) and rs1051730 cholinergic receptor nicotinic alpha 3 subunit (CHRNA3) were associated with lower odds for cessation (odds ratio (OR) 0.88, 95% confidence interval (CI) 0.80–0.97 (*n* = 5 cohort studies)). The authors concluded that SNPs rs16969968/rs1051730 may be considered as risk alleles for persistent smoking [6]. The effect of different cytochrome P450 (CYP) 2A6 genotypes (slow, intermediate or normal metabolizers) in terms the ability to quit smoking also supports nicotine’s central role in smoking cessation. CYP2A6 is responsible for the metabolism of xenobiotics, including nicotine. Conversely it was found that people with lower enzyme activities (slow metabolizers) are more likely to quit smoking [7]. For example, in a study of 308 Caucasian adolescent smokers the odds ratio for smoking cessation was 2.25 (95% CI 1.05–4.80; *P* = 0.037) for slow metabolizers relative to normal metabolizers. The authors posited the influence of genetic variation regarding smoking cessation [8].

The addictive potential of nicotine stems from its effect on the dopaminergic reward system [9, 10]. Activation of the nicotinic acetylcholine receptor increases dopamine transmission by stimulating dopamine release and reuptake. This effect is modulated by noradrenaline neurotransmission, as dopaminergic and noradrenergic neurons in the nucleus accumbens act synergistically in terms of their roles in reward signaling [11]. Dopamine is predominantly catabolized by catechol-o-methyltransferase (COMT) in the extracellular space by O-methylation, while noradrenaline undergoes oxidative deamination by monoamine oxidase A (MAO-A) found intracellularly in the presynaptic terminal of monoaminergic neurons and extrasynaptically in astrocytes [12]. The metabolic enzymes COMT and MAO-A are known to have functional polymorphisms (e.g. for COMT rs4680; for MAO-A rs6609257, and rs6323, respectively) [13–15]. The SNP in the coding region of COMT at position 158 (rs4680; minor allele adenine (A), major allele guanine (G)) leads to the amino acid substitution of methionine (Met) for valine (Val) resulting in up to 75% reduction of enzymatic activity [16, 17]. Association of smoking with rs4680 COMT polymorphism was reported among men, as differences in G/G (24.4% vs. 23.4%), G/A (42.8% vs. 54%) or A/A (32.8% vs. 22.6%) genotype frequencies differed significantly (*P* = 0.017) between male smokers and nonsmokers, respectively [13]. Similarly, an SNP in the coding region of the X-linked MAO-A gene at position 941 (rs6323, minor allele A, major allele G) results in a 75% reduction of MAO-A enzyme activity in homozygotic females and hemizygotic males when compared to those with only G alleles [15, 18].

The objective of the current study was to attest whether the presence of the minor allele A for rs4680 and rs2235186 polymorphisms of COMT and MAO-A, respectively are associated with the ability to quit smoking (e.g. presence of lower odds for smoking cessation with higher minor allele load) among chronic inflammatory pulmonary disease patients.

## Methods

### Study design and protocols

The present study was planned and implemented in line with the STROBE statement for cross-sectional studies [19]. It was approved by the National Center for Public Health (Hungary) and its expert committee - the Human Reproduction Committee of the Hungarian Medical Council (IF12617-9/2015). Informed consent was obtained from each participant. The investigation conforms to the principles outlined in the Declaration of Helsinki.

The current study is based on the analysis of the cohort of patients who attended the outpatient unit of the Department of Pulmonology (University of Debrecen, Hungary) between January 4. 2016 and December 31. 2017 for the management of chronic airway inflammatory diseases (BA, COPD). Inclusion criteria for participation were as follows: the patient was older than 18 years of age at inclusion, was registered at the Department with the diagnosis of BA or COPD, was a current smoker or smoked previously and was able to give informed consent. Exclusion criteria were limited to age below 18 years, and unavailability of informed consent. Each patient who was able to give his/her consent after a thorough discussion with the research physician was invited to participate. Overall, 152 patients with a current smoking habit or history of smoking were included in the current analysis.

At the time of recruitment, patients were already diagnosed with BA or COPD and were managed according to the relevant GOLD [20] and GINA [21] initiatives. Patients received therapy at the time of inclusion as clinically warranted. Demographic, socioeconomic (family status, number of children, highest level of education, number of people living in the household, employment status, type of work (physical or intellectual work), anamnestic, and laboratory data were acquired. The Fagerstrom questionnaire was administered only if the patient was a smoker at the time of the study [22]. Information regarding current or past smoking, attempts of, success of smoking cessation, number of smokers closely associated with the patients (e.g. of the 3 to 10 people you spend most of your time with how many smokes, and how many doesn’t smoke?) were also documented. Methods supporting smoking cessation attempts were recorded. Success of cessation was defined using a dual criterion of self-reports and urine cotinine levels, accordingly smoking cessation was considered successful if the patient previously smoked but at the time of our investigation was a self-reported nonsmoker with urine cotinine levels were below 550 ng/ml [23].

### Blood sampling and DNA extraction

Blood was collected from the cubital vein on the morning of the examination. Genomic DNA was extracted from peripheral blood specimens according to standard protocols using the Genomic DNA Purification Kit (Thermo Fisher Scientific #K0512). The quality and integrity of DNA were evaluated by agarose gel electrophoresis and UV-spectrometry. Based on the image of agarose gel electrophoresis DNA samples were intact.

### PCR amplification and DNA sequencing for genes MAO-A and COMT

SNPs, rs4680 COMT polymorphism and rs2235186 MAO-A polymorphism were genotyped using TaqMan SNP genotyping assays with probes C_25746809_50 and 4,351,379 C__22272420_10, respectively (Applied Biosystems, Foster City, CA). Following polymerase chain reaction (PCR) amplification, the endpoint fluorescence was read with the ABI QuantStudio 12 K Flex instrument and genotypes were assigned using the allelic discrimination option of QuantStudio 12 K Flex Software v1.2.2 (Applied Biosystems, Foster City, CA). The average genotyping success rate of at least 95% was attained for both SNPs.

### Urine samples and cotinine determination

Routine laboratory investigation for urine creatinine levels were done according to the standard laboratory protocol of the Department of Laboratory Medicine (University of Debrecen). Urine cotinine levels descriptive of nicotine consumption were obtained by GC-MS as follows. Urine samples were thawed and centrifuged at 1 700 RPM (10 min, 18 ºC). Supernatants were diluted with highly purified distilled water according to the smoking status of the patients, samples were diluted by factor 40 and 20 in case of regular smokers vs. occasional smokers, respectively. Samples of current nonsmokers were not diluted. Urine cotinine levels were determined using Immulite 2000 autoanalyzer (DPC: Diagnostic Product Corporation, Los Angeles, USA. Cat no: L2KNM2)). Nicotine Metabolite Adjustors were used for calibration, Serum Drug Control Medium (SDCM) was used for control. Every control was within the range of reference provided by the manufacturer. The range of detection for cotinine was between 10 and 500 ng/ml. If the measured value exceeded the > 500 ng/ml upper threshold, the measurement was repeated after further diluting the sample. If the measured value was below the limit of detection (< 10.0 ng/ml) the measurements were repeated with the undiluted sample. Patients were considered smokers if they reported themselves as smokers or if they were self-reported nonsmokers with urine cotinine levels above 550 ng/ml [23].

### Statistical analysis

Normality of continuous variables was checked by the Shapiro-Wilk test. Continuous variables were not normally distributed, hence Mann-Whitney U test was carried out to compare two data sets. Frequencies were compared by the Pearson’s χ^2^ test or Kruskal-Wallis test. Demographic, sociodemographic, anamnestic, laboratory (urine cotinine levels) and allele frequencies for COMT and MAO-A polymorphisms were compared with respect to the type of chronic inflammatory airway disease (AB vs. COPD) and success of smoking cessation. Basic demographic and clinical parameters were compared with respect to the distinct genotypes of the two polymorphisms. Hardy-Weinberg equilibrium was calculated using the equation: p^2^ + 2rp + r^2^ = 1 (with p and r denoting allele frequencies), for the whole sample and the subset of women for rs4680 COMT and rs2235186 MAO-A polymorphisms, respectively using STATA 13 (USA) [24].

Multiple logistic regression was performed to assess the potential contribution of rs4680 COMT and rs2235186 MAO-A polymorphisms to the ability to quit smoking. First simple logistic regression analysis was done with possible determinants of smoking cessation (number of people living in the patients’ household, level of education, employment status, family status, number of smokers among close acquaintances). Additionally, the possible mediating role of disease type and duration of the disease was assessed by simple logistic regression. Missing data were omitted. After univariate testing all significant regressors as well as age and sex (as a priori variables) were entered into a multiple logistic regression model to further characterize the relationship between success of smoking cessation and selected polymorphisms. For the dominant models the heterozygotes were considered to have the high activity phenotype of COMT characteristic of the G/G genotype thus G/G and G/A genotypes were compared to A/A genotypes, while under the assumption of recessive inheritance heterozygotes were deemed to have the low activity phenotype of COMT, thus G/A and A/A genotypes were pooled. Due to the fact that some reported a codominant inheritance mode for rs4680 [25] while others analyzed clinical data under the assumption of dominant or recessive inheritance [26, 27], all three – dominant, recessive and additive inheritance models were run. As for rs2235186 polymorphism, it was reported that heterozygotes have intermediate levels of MAO-A activity with females homozygous with 2 A (minor) alleles or males hemizygous with one allele showing 75% lower enzyme activity [15], hence an additive model was implemented [14]. The initial model contained the two polymorphisms, age, sex, the total number of years when the patient smoked, level of education (as a binary variable high school diploma or higher vs. no high school diploma), the number of smokers among the 3 to 10 closest acquaintances (as a binary variable most nonsmoker/most smoker or equal number of smokers and nonsmokers), number of people living in the household of the patient. Variables were introduced into the model simultaneously, followed by omission of the non-significant factors (e.g. those not contributing significantly to the model). Goodness of fit for the model was assessed by Hosmer-Lemeshow goodness-of-fit test. Statistical analysis was performed with Stata 13.0 software (Stata Corporation). Values are given medians (with interquartile ranges: IQR), and odds ratios presented with their 95% confidence intervals.

## Results

### Patients

Of the 152 patients included 74 (48.68%) and 78 (51.32%) patients were females and males, respectively, the median age of the sample was 68 years (IQR 61–75 years) and our sample included 29 BA and 123 COPD patients. Disease duration varied with respect to the type of disease with medians of 20 years (IQR 9–40 years) and 5 years (IQR 2–14 years) for patients suffering from BA and COPD, respectively (*p* < 0.01). At the time of assessment 64 (42.10%) patients were smokers (37 (24.34%) female and 27 (17.76%) male patients), while 88 (57.90%) patients were former smokers (37 (24.34%) female and 51 (33.56%) male patients). The median duration of smoking years was 34.5 years (IQR 20–40 years). Among current smokers the median of the total score for Fagerström test for nicotine dependence was 5 points (IQR 4–6 points). Frequency of smoking patients did not differ significantly with respect to underlying pulmonary disease (*p* = 0.61). Seven smokers reported the use of pharmacological therapy to support their smoking cessation attempt with 6 patients using nicotine replacement therapy and one patient using varenicline. Each of these 7 patients were smokers at the time of the current study. Characteristics of the patient population are shown in Tables 1 and 2.

**Table 1:** Characteristics of the per analysis set of patients with chronic inflammatory pulmonary disease who smoke or previously smoked (*n* = 152) and of its two subgroups regarding success of smoking cessation (successfully quit smoking (*n* = 88) and failed to quit smoking (*n* = 64). The f and m regarding p values denote the level of significance with respect the differences of allele frequencies among female and male patients, respectively (bold p values represent the statistically significant (*p* < 0.05) results)

Parameters	Whole population (*n* = 152 patients)	Successfully quit smoking (*n* = 88 patients)	Failed to quit smoking (*n* = 64 patients)	*p*
Age (years)	68 (61–75)	71 (64-78.5)	64 (58.5–69.5)	**< 0.001**
Gender (f/m)	74/78	37/51	37/27	0.055
Smoker (n/y)	88/64	88/0	0/64	**< 0.001**
Smoking (years)*	34.5 (20–40)	30 (11–40)	37 (29–45)	**0.004**
Urine cotinine (ng/ml)	89.85 (9.9–2114)	9.9 (9.9–21.3)	3171 (758.5-5175.5)	**< 0.001**
Disease type (AB/COPD)	29/123	18/70	11/53	0.613
Disease duration (years)	9 (2–16)	8.5 (2–15)	9.5 (3–20)	0.486
Level of education (high school graduate or higher/no high school diploma)	33/118	25/63	8/55	**0.021**
Employment (n/y)	132/19	80/8	52/11	0.126
Family status (in relationship/single)	87/64	46/42	41/22	0.116
Number of smokers among close acquaintance (most nonsmoker/most smoker or equal number of smokers and nonsmokers)	112/38	75/13	37/25	**< 0.001**
number living in household (people)	2 (1–2)	2 (1–2)	2 (1–3)	0.123
MAO-A (rs2235186) AA (f/m) AG (f/m) GG (f/m)	12/22 34/- 28/55	7/15 16/- 14/35	5/7 18/- 14/20	**< 0.001**
COMT (rs4680) AA AG GG	32 77 43	14 45 29	18 32 14	*p* = 0.12

* smoking in years for current smokers and past smoking years for those who successfully quit smoking

**Table 2:** Characteristics of the per analysis population of AB and COPD patients who previously or currently smoked (*n* = 152) and of its two subgroups regarding the type of chronic inflammatory airway disease (AB (*n* = 29) and COPD (*n* = 123)). The f and m regarding p values denote the level of significance with respect the differences of allele frequencies among female and male patients, respectively (bold p values represent the statistically significant (*p* < 0.05) results)

Parameters	AB (*n* = 29 patients)	COPD (*n* = 123 patients)	*p*
Age (years)	64 (56–70)	69 (62–75)	**0.022**
Gender (f/m)	23/6	51/72	**< 0.001**
Smoker (n/y)	18/11	70/53	0.613
Smoking (years)*	20 (5–30)	35 (22–45)	**< 0.001**
Urine cotinine (ng/ml)	46.9 (9.9–706)	176 (9.9–2268)	0.238
Successful cessation (y/n)	18/11	70/53	0.613
Disease duration (years)	20 (9–40)	5 (2–14)	**< 0.001**
Level of education (high school graduate or higher/no high school diploma)	7/22	26/96	0.74
Employment (n/y)	22/7	110/12	**0.037**
Family status (in relationship/single)	15/14	72/50	0.47
Number of smokers among close acquaintance (most nonsmoker/most smoker or equal number of smokers and nonsmokers)	21/8	91/30	0.756
number living in household (people)	2 (2–4)	2 (1–2)	**0.029**
MAO-A (rs2235186) AA (f/m) AG (f/m) GG (f/m)	4/0 10/- 9/5	8/22 24/- 19/50	0.146
COMT (rs4680) AA AG GG	5 12 12	27 65 31	**0.022**

The minor allele is A for both rs2235186 MAO-A and rs4680 COMT polymorphisms, respectively. The rs4680 was in Hardy-Weinberg equilibrium regarding the whole sample (*p* = 0.872). The rs2235186, the X chromosome-linked SNP was also in Hardy-Weinberg equilibrium among the female patients (*p* = 0.808). The basic demographic and clinical factors regarding GG, AG and GG genotypes for rs2235186 MAO-A and rs4680 COMT polymorphisms are presented in the Supplementary Table 1 (see Supplementary material).

### Comparison of patients with respect to success of smoking cessation

Data of the 152 patients was compared with respect to success of smoking cessation. The two groups proved to be homogenous regarding the distribution across sex (males vs. females), type of inflammatory airway disease (BA vs. COPD), employment state (employed vs. unemployed), family status (living in a relationship, vs. living alone). However, there were numerous statistically significant differences between the two subgroups (those who quit smoking vs. who did not). Patients who successfully quit smoking were older (71 (IQR 64-78.5 years vs. 64 (IQR 58.5–69.5) years; *p* < 0.001), smoked for fewer years (30 (IQR 11–40) years vs. 37 (IQR 29–45) years; *p* = 0.004). Higher proportion of successful quitters had at least a high school diploma (28.41% vs. 12.5% for success vs. no success quitting, respectively; *p* = 0.021). Furthermore, of the 3 to 10 people with whom the patients spent most of their time, those who successfully quit were surrounded more frequently by nonsmokers than those who were unable to put down the cigarette (*p* < 0.001). Allele frequencies of rs4680 COMT showed significant difference (*p* = 0.01) among the subset of male patients, in terms of success for quitting or failing to quit smoking (Table 1).

### Comparison of patients with respect to the underlying disease pulmonary disease

Assessing the data with respect to the underlying disease showed that patients with BA were significantly younger (64 (IQR 56–70) years vs. 69 (IQR 62–75) years for BA vs. COPD, respectively, *p* = 0.022), and had a shorter course of smoking in their case history (20 (IQR 5–30) years vs. 35 (IQR 22–48) years for BA vs. COPD, respectively, *p* < 0.001), paralleled by a longer course of disease (20 (IQR 9–40) years vs. 5 (IQR 2–14) years for BA vs. COPD, respectively, *p* < 0.001). The proportion of unemployed patients was lower among patients with BA who lived in bigger households than did COPD patients. (Table 2).

### Significant regressors for successful smoking cessation

Upon assessing the effect rs4680 COMT polymorphism regarding the success of smoking cessation a significant relationship was identified for the additive model (*p* = 0.044), while the relationship was on the verge of significance in the dominant model (*p* = 0.071) and it was not significant in the recessive model (*p* = 0.137). There was a lack of significant association regarding MAO-A rs2235186 and smoking cessation (Table 3).

**Table 3:** Regressors of the success for smoking cessation determined with simple logistic regression (*n* = 152). Significance levels *p* < 0.05 are indicated in bold

Predictor variable	Odds ratio (95% CI)	*p*
**Simple logistic regression for successful cessation**		
COMT (rs4680) dominant	0.48 (0.22, 1.06)	0.071
COMT (rs4680) additive	0.61 (0.38, 0.98)	**0.044**
COMT (rs4680) recessive	0.57 (0.27, 1.19)	0.137
MAO-A (rs2235186) dominant	1.47 (0.66, 3.24)	0.343
MAO-A (rs2235186) additive	1.05 (0.71, 1.56)	0.804
MAO-A (rs2235186) recessive	0.87 (0.46 1.68)	0.696
Age (years)	1.08 (1.04, 1.12)	**< 0.001**
Sex (f/m)	1.89 (0.98, 3.63)	0.056
Disease type (AB/COPD)	0.81 (0.35, 1.85)	0.613
Disease duration (years)	1.00 (0.98, 1.03)	0.82
Education (high school graduate or higher/no high school diploma)	0.37 (0.15, 0.88)	**0.024**
Number living in household (people)	0.76 (0.59, 0.98)	**0.039**
Employment (y/n)	1.71 (0.54, 5.35)	0.358
Family status (in relationship/single)	1.70 (0.87, 3.21)	0.118
Smoking years (years)	0.96 (0.94, 0.99)	**0.003**
Number of smokers among close acquaintance (most smoker or equal number of smokers and nonsmokers/ most nonsmoker)	3.89 (1.79, 8.48)	**0.001**

After adjusting for all significant regressors and a priori determinants by means of multiple logistic regression, the significant effect of COMT rs4680 became more pronounced and reached statistical significance in all three models, e.g. the dominant, additive and recessive models with odds ratios of 0.22 (0.079, 0.63; *p* = 0.005), 0.37 (0.20, 0.69; *p* = 0.002), and 0.34 (0.13, 0.89; *p* = 0.028), respectively (Table 4). This means that presence of minor allele A is accompanied by decreased odds for success of smoking cessation. The effect of MAO-A rs2235186 failed to reach statistical significance. Nonetheless, the odds ratios were higher than one, ranging between 1.23 and 1.31, suggesting a trend for successful smoking cessation with higher minor allele load. The Hosmer-Lemeshow goodness-of-fit test showed good fit for all three models (*p* = 0.150, *p* = 0.347 and *p* = 0.174, for the dominant, additive and recessive models, respectively).

**Table 4:** Final multiple logistic models for the success for smoking cessation (*n* = 152). All three possible inheritance models (dominant, additive and recessive) for the rs4680 COMT polymorphism are shown. (number of people living in the patients’ household is included in all three models, as its exclusion form the models led to significantly different nested models.) Significance levels *p* < 0.05 are indicated in bold

Predictor variable	Odds ratio (95% CI)	*p*
**Dominant model for COMT (rs4680)**		0.150
COMT (rs4680)	0.22 (0.079, 0.63)	**0.005**
MAO-A (rs2235186)	1.31 (0.76, 2.24)	0.331
Age (years)	1.11 (1.05, 1.18)	**< 0.001**
Sex (f/m)	3.12 (1.31, 7.45)	**0.010**
Smoking years (year)	0.93 (0.89, 0.96)	**< 0.001**
Number of smokers among close acquaintance (most smoker or equal number of smokers and nonsmokers/ most nonsmoker)	3.12 (1.24, 7.89)	**0.016**
Number living in household (people)	0.83 (0.59, 1.17)	0.287
**Additive model for COMT (rs4680)**		0.347
COMT (rs4680)	0.37 (0.20, 0.69)	**0.002**
MAO-A (rs2235186)	1.28 (0.74, 2.23)	0.376
Age (years)	1.11 (1.05, 1.18)	**< 0.001**
Sex (f/m)	3.51 (1.43, 8.61)	**0.006**
Smoking years (year)	0.93 (0.89, 0.96)	**< 0.001**
Number of smokers among close acquaintance (most smoker or equal number of smokers and nonsmokers/ most nonsmoker)	2.99 (1.18, 7.59)	**0.021**
Number living in household (people)	0.80 (0.57, 1.28)	0.204
**Recessive model for COMT (rs4680)**		0.174
COMT (rs4680)	0.34 (0.13, 0.89)	**0.028**
MAO-A (rs2235186)	1.23 (0.71, 2.13)	0.460
Age (years)	1.11 (1.05, 1.17)	**< 0.001**
Sex (f/m)	4.07 (1.62, 10.28)	**0.003**
Smoking years (year)	0.93 (0.90, 0.96)	**< 0.001**
Education (no high school diploma/high school graduate or higher)	3.22 (1.09, 10.00)	**0.035**
Number of smokers among close acquaintance (most smokers or equal number of smokers and nonsmokers/ most nonsmoker)	2.47 (0.99, 6.17)	0.053
Number living in household (people)	0.88 (0.62, 1.23)	0.478

All three final multiple logistic regression models for successful cessation showed increased odds for success with each additional life year. On the contrary each additional year of smoking in the case history of patients decreased the odds for successful smoking cessation. Male sex, higher number of nonsmokers among closest acquaintances significantly increased the odds for successful smoking cessation as well. Furthermore, in the recessive model having at least a high school diploma was associated with higher odds for success of smoking cessation (3.22 (1.09, 10, *p* = 0.035) (Table 4).

## Discussion

Presence of the minor allele A for rs4680 COMT polymorphism was associated with significantly lower odds for successful smoking cessation in a population likely to be exposed to smoking cessation initiatives, e.g. patients receiving maintenance therapy for chronic inflammatory airway disease. Accordingly, A/A subjects had lower odds for success (0.37; 95% CI 0.2–0.69) regarding smoking cessation when compared to G/G, or G/A patients (additive model). Similar results were found under the assumption of dominant and recessive inheritance modes with odds ratios for successful cessation being 0.22 (CI 0.08–0.63) and 0.34 (CI 0.13–0.89), respectively. On the other hand, patients homozygous for the minor allele (A) at rs2235186 of MAO-A showed a trend toward increased odds for success regarding smoking cessation (albeit this failed to reach statistical significance). This effect was present in all three final models (the additive, dominant and recessive model for rs4680 COMT polymorphism). Furthermore, we found that patients were more likely to quit smoking as they got older, while success to quit was less likely if patients smoked for a longer time. Interesting to note that the odds for smoking cessation is higher among men and increases if the most of the closest acquaintances of the patient are nonsmokers, suggesting the role for social inclusion and susceptibility to negative social cues in smoking behavior [14, 28, 29].

To the best of our knowledge the current study is the first to assess the association of COMT and MAO-A polymorphisms and smoking cessation among patients managed for chronic inflammatory airway disease. Our results are consistent with the results of other larger genetic studies, which have reported similar results in different populations. In a population-based cohort study of the elderly (> 55 years old) retrospective and prospective analysis showed that G/G homozygote genotype is associated with higher likelihood of smoking cessation [27]. The results of this prospective analysis are very robust, as 1 195 patients were followed for 12 years. The authors reported significantly lower odds ratio for success (OR 0.7., 95% CI: 0.55–0.88, *p* = 0.003) when G/G genotype was compared with A/A and G/A genotypes, in pooled analysis of incident and prevalent cessation cases vs. persisting smokers. Conversely, a case-control study of women of European ancestry (*n* = 541) showed increased odds for being a former regular vs. current regular smoker if the minor allele was present (OR of 1.45; 0.92–2.30 and 1.82; 1.05–3.17 for G/A and A/A genotypes compared to the G/G genotype, respectively, *p* = 0.03) [26]. Similar findings were reported regarding smokers enrolled in clinical trials. Accordingly the A allele was associated with lower likelihood of short-term and long-term abstinence (relative risk reduction (RRR) 0.9; 95% CI 0.6–1.5 and RRR 0.7; 95% CI 0.4–1.3, respectively, for women with A/A genotypes vs. G/G genotypes for the rs4680 COMT gene) in the placebo arm of a d, l-fenfluramine replacement trial [30]. Likewise, higher frequency of the G/G genotype was found in the abstinence group than the nonabstinence group (χ^2^ = 8.12, *p* = 0.02 for the G/G genotype) in the placebo arm of a buproprion trial [31]. Furthermore, systematic review of combined sex studies showed significantly lower odds for the association between the minor allele (A) and smoking cessation (summary OR: 0.73; 95% CI 0.57–0.93, *n* = 3 studies) [32]. Nonetheless there are studies which failed to show a significant association between smoking cessation and rs4680 COMT polymorphism [33].

**Fig. 1:**
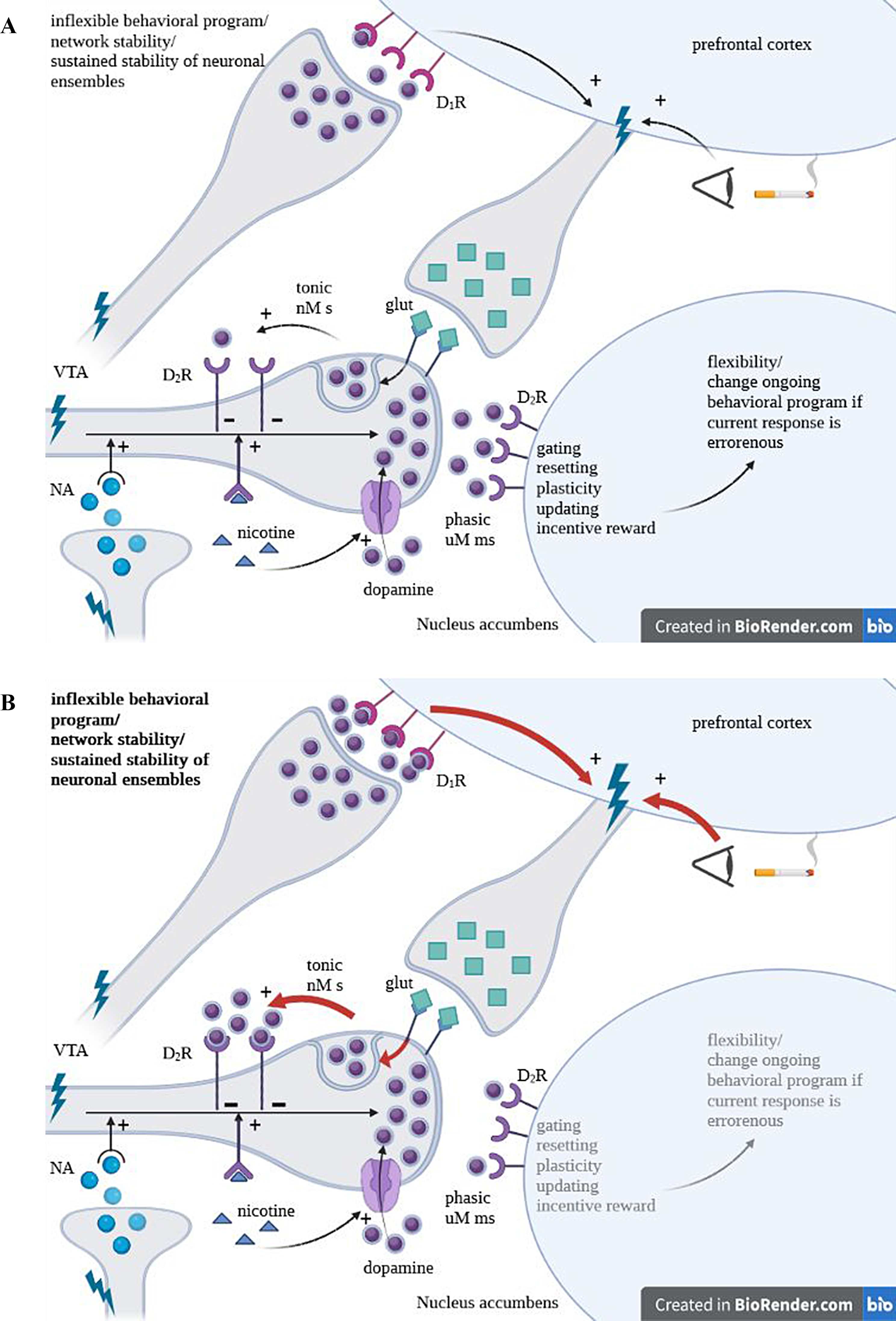
(**A**) Possible mechanism underlying the failure of cessation attempts in Rs4680 G/A COMT and rs2235186 polymorphism. Panel A: Activation of ventral tegmental area (VTA) dopaminergic neurons leads to the phasic release of dopamine in the nucleus accumbens (NAcc), and the prefrontal cortex. VTA dopaminergic neurons may be activated by noradrenergic neurons and nicotine, both enhancing phasic dopamine release. Phasic release of dopamine is also seen as a consequence of action potential discharge, and results in postsynaptic dopamine receptor activation. Tonic dopamine release on the other hand is determined by the intrinsic pacemaker-like membrane currents of dopaminergic neurons leading to spontaneous baseline spike activity [34]. Increased dopaminergic tone selectively activates high affinity D2 receptors, located on the presynaptic neurons. Presynaptic D2 receptor activation has an autoregulatory role as it downregulates phasic dopamine release. Thus, excessive tonic dopamine levels, by inhibiting phasic dopamine burst firing [16], skew the tonic/phasic balance. This alters reward-directed behavior [35], and decreases reward-related activation in the mesolimbic system. Glutamatergic input of the NAcc from the prefrontal cortex (that may be activated by visual input) leads to increased extrasynaptic dopamine release that by activating presynaptic D_2_ receptors inhibits phasic firing of the dopamine neurons. Dopamine in the NAcc is cleared from the synapse by presynaptic dopamine transporters (DAT), a mechanism that is considerably more limited in the prefrontal cortex placing greater emphasis on the function of metabolic enzymes COMT and MAO-A. (**B**) Rs4680 G/A COMT polymorphism leads to decreased activity of the enzyme leading to sustained dopamine action. This is more pronounced in the prefrontal cortex, where elimination of synaptic dopamine is done by enzymatic degradation due to the sparse availability of DAT. As a result, activation of the prefrontal glutamatergic neurons by dopamine input leads to more pronounced release of extrasynaptic dopamine, causing the inhibition of phasic dopamine release in the NAcc (these changes are reflected by the red arrows). Lack of phasic dopamine release contributes to nicotine seeking behavior, susceptibility to smoking related cues. Furthermore, heightened prefrontal cortex activity favors inflexible behaviors and network stability, paralleled by diminished plasticity and flexibility of change in behavior developing due to diminished phasic dopamine release in the NAcc [16, 42, 43]. These factors may influence the ability to be persistent regarding the attempt to quit smoking, as seen in the current study. Furthermore, smoking or nicotine replacement, by enhancing the phasic component of dopaminergic neuronal activity restores the balance between tonic and phasic dopamine release and effect especially favorable in patients with A/A alleles. Thus, patients with A/A genotypes may seek to smoke, so they are less likely to adhere to their intention to quit smoking to maintain their wellbeing, or alternatively may be more responsive to nicotine replacement therapy

COMT and MAO-A play a central role in monoamine metabolism including dopamine, noradrenaline and serotonin, hence any functional alteration of these two enzymes leads to the change of the level of these catecholamines [9]. The effect COMT polymorphism in the context of smoking cessation must be addressed by considering the differential role of COMT in the nucleus accumbens and the prefrontal cortex (PFC) regarding dopamine metabolism [16, 34, 35]. Furthermore, change in striatal dopamine levels must be assessed considering the balance between tonic/phasic dopamine release [34]. Our finding of lower cessation success for A/A alleles is in agreement with this conceptualization, as A/A genotype is associated with lower enzyme activity, leading to increased tonic dopamine levels. Omission of burst firing may lead to craving and drug-seeking behavior [35], a phenomenon that could underscore lower cessation success (Fig. 1A). It is proposed that patients with higher A allele load are less likely to quit smoking due to elevated tonic dopamine levels which in turn blunts the reinforcing phasic dopamine signal resulting in dysphoria. Lack of reward increases the drive to restore normal phasic/tonic balance for example by reinstating nicotine consumption (Fig. 1B).

The complex interplay between dopaminergic neurons and nicotine has been described previously. Nicotine enhances dopamine reuptake into the presynaptic neuron increasing dopamine concentration [9, 36], and binds to nicotinic acetylcholine receptors, increasing phasic activity of mesolimbic and mesocortical dopaminergic neurons [3]. Accordingly studies show better treatment response with nicotine replacement therapy paralleled by increased A allele load. In a prospective cohort study, patients who attempted to quit smoking received nicotine transdermal patch or placebo for twelve weeks. The authors found higher likelihood of abstinence at 12-week follow-up, with 33% vs. 12%, of patients with the COMT A/A genotype showing abstinence while receiving the transdermal patch vs. placebo treatment, respectively as opposed to 22% vs. 16% of patients being abstinent if COMT G/A or G/G genotype was present (*p* < 0.001). Additionally, significant genotype x treatment effect was observed at 12-week follow-up (OR 0.43, 95% CI 0.19-1.00; *p* = 0.05) [37]. In a randomized, open-label smoking cessation trial enrolling 178 women of European ancestry, women homozygous for the A allele were more likely than women with G/G genotype to be abstinent from smoking at end of treatment 8 weeks long nicotine replacement therapy (OR 2.96, 95% CI 1.07–8.14 for A/A genotype and OR 1.26 95% CI 0.5–3.22 for G/A genotype, *p* = 0.04) [26]. The above findings offer a plausible explanation for our results, showing lower odds for successful cessation in patients with minor (A) allele genotypes for rs4680 COMT leading to lower enzyme activity that presumably contributes to increased tonic and decreased phasic dopamine activity in the nucleus accumbens.

A further implication of the rs4680 G/A polymorphism concerns its effect in the prefrontal cortex, where COMT is the key enzyme for metabolizing synaptic dopamine given the relative lack of dopamine transporters [38]. Thus, here the rs4680 A allele corresponds with increased tonic dopamine levels. This has several implications regarding executive functions, including orientation of attention [39], attentional set shifting [40]. Given the known attentional bias for nicotine-related stimuli in smokers [41], increased prefrontal function, including orientation and shifting attention could lead to prompt identification of smoking-related cues in rs4680 A allele carriers and homozygotes. Craving-induced cues, by activating cortico-accumbent glutamatergic neurons will increase the tonic dopamine levels in nucleus accumbens, further decreasing the reinforcing phasic dopamine signal [35]. This, by skewing the phasic/tonic dopamine balance may induce craving and nicotine seeking behavior, leading to the failure of smoking cessation attempts.

A genome wide association study implicated the role of functional polymorphism of MAO-A regarding distinct smoking related behaviors. The SNP rs2235186 of MAO-A gene was identified as a candidate gene at *p* < 0.01, regarding two continuous variables descriptive of smoking behavior e.g. the number of cigarettes per day and pack-years [44]. No additional studies were identified concerning the possible association between rs2235189 MAO-A polymorphism and smoking cessation per se. Nonetheless, others have described the potential role of this SNP in other behavioral domains. The low activity form of the enzyme was implicated in experiencing greater emotional reactivity to negative social cues [11]. Similarly, others have reported an increase in self-reported hypersensitivity after social exclusion among individuals with the low expression allele of MAO-A gene [45]. In the context of chronic inflammatory pulmonary disease smoking is considered untoward, as it fuels the progression of the disease, and limits the patient’s quality of life [46]. Regular counselling by healthcare personnel, expectations of the family members and significant others all point toward the need to quit, an attitude that may transpire as social exclusion or social rejection if smoking is continued. The trend of higher odds for successful smoking cessation (albeit not a statistically significant finding) paralleled by the increased load of the minor allele for rs2235186 MAO-A polymorphism may be interpreted in this social context. Lower MAO-A enzyme activity, by contributing the higher sensitivity to social exclusion may motivate individuals to quit smoking. Susceptibility for social cues is also emphasized by our finding of higher prevalence of smokers around those patients who were unable to quit smoking (lower odds for smoking cessation). This finding is in line with reports of others. In a study of 2 431 smokers, social smoking cue was characterized by the question “other than yourself does anyone regularly smoke cigarettes in your presence, such as at your home, work, car, or other places?”. The authors found that social smoking cue is significant factors for relapse [47].

The present study has some limitations. COMT and MAO-A are involved in the metabolism of several monoamines including dopamine, noradrenaline and serotonin. Albeit the role of altered dopaminergic tone was articulated in the present study, the complex interplay between all three neurotransmitters at the nucleus accumbens, and the prefrontal cortex may be responsible for some of the findings presented here. Direct assessment of dopamine, noradrenaline and serotonin levels were omitted in the current study, due to budgetary constraints. Additionally, the possible influence of pharmacological supportive therapy for smoking cessation was not considered in the analysis, as the majority (95.4%) of patients did not use pharmacological support. Nevertheless, the 7 patients who used pharmacological treatment were not successful with their cessation attempts, hence all of them were smokers at the time of the current study. Furthermore, the current study lacked data regarding disease severity or general well-being, factors that may also contribute to the outcome of smoking cessation efforts. Additionally, no data was available regarding duration of smoke-free status of patients who quit smoking. Validation with similar cohorts is needed in future studies.

Nevertheless, the current study has several merits. Selection and information bias was avoided since patients were enrolled consecutively as they attended regular care for their chronic pulmonary disease, without prior knowledge regarding their genotypes or smoking habits. Misclassification bias was minimized by a dual approach to define success of smoking cessation, accordingly self-reported smoking abstinence and a predefined urine cotinine threshold of 550 ng/ml was applied. Furthermore, using a multiple logistic model possible confounding factors (age, sex, type of pulmonary disease, disease duration and proportion of smokers among close acquaintances) were corrected for.

In conclusion, the present study offers new insight into the influence of functional genetic polymorphisms on the success of smoking cessation in the context of chronic inflammatory pulmonary disease. This population is special in terms of smoking given the causal role in disease progression. Presence of the minor allele for rs4680 COMT was shown to decrease the odds for successful smoking cessation, a finding that may be interpreted in view of the altered balance between tonic and phasic dopamine release. The consequently increased tonic and decreased phasic dopamine release in the nucleus accumbens may be restored by nicotine consumption, thus impeding attempts to quit smoking. Thus, it should be note that regardless the ongoing medical care received for chronic inflammatory pulmonary disease success of smoking cessation was related to genetic polymorphism of COMT, suggesting the need for identifying targeted pharmacogenomic approaches in this patient population. In addition, the current study offers a comprehensive framework for understanding the neurobiological and behavioral aspects regarding the change of tonic dopamine levels in the nucleus accumbens and prefrontal cortex and suggests the role of executive and cognitive constructs influenced by these polymorphisms in determining the success of cessation attempts.

### Electronic supplementary material

Below is the link to the electronic supplementary material.

Supplementary Material 1

## Data Availability

Further information is available on request from the corresponding author.
