# Supplementary material for: Association of monoaminergic gene polymorphisms in chronic inflammatory pulmonary disease patients with successful smoking cessation
Source: BMC Pulm Med. 2024 Aug 26;24:411. doi: 10.1186/s12890-024-03219-y (PMC11348745; doi:10.1186/s12890-024-03219-y)
Supplement: Supplementary file 1 — Supplementary Material 1 [file 12890_2024_3219_MOESM1_ESM.docx]

Supplementary Table 1: basic demographic variables and clinical factors with respect to rs2235186 MAO-A and rs4680 COMT polymorphisms; significance levels p<0.05 are indicated in bold

| **rs2235186** | **GG** | **AG** | **AA** | **p** |
| --- | --- | --- | --- | --- |
| age (years) | 69 (63-77) | 64.5 (60-71) | 67.5 (58-75) | 0.10 |
| sex (f/m) | 28/55  33.7%/66.3% | 34/0  100%/0% | 12/22  35.3%/64.7% | **<0.01** |
| smoker (n/y) | 49/34  59.0%/41.0% | 16/18  47.0%/53.0% | 22/12  64.7%/35.3% | 0.31 |
| disease type (AB/COPD) | 14/69  16.9%/83.1% | 10/24  29.4%/70.6% | 4/30  11.8%/88.2% | 0.15 |
| level of education (high school graduate or higher/no high school diploma) | 18/65  22.6%/78.4% | 10/24  29.4%/70.6% | 4/29  12.1%/87.9% | 0.22 |
| employment (n/y) | 74/9  89.1%/10.9% | 28/6  82.3%/17.7% | 29/4  87.8%/12.2% | 0.60 |
| family status (in relationship/single) | 48/35  57.8%/42.8% | 15/19  44.1%/55.9% | 23/10  69.7%/30.3% | 0.11 |
| number of smokers among close acquaintance (most nonsmoker/most smoker or equal number of smokers) | 64/18  78.0%/22.0% | 23/11  67.7%/32.3% | 24/9  73.7%/27.3% | 0.49 |
| **rs4680** | **GG** | **AG** | **AA** | **p** |
| age (years) | 65 (60-78) | 68 (61-75) | 68.5 (63.5-72) | 0.95 |
| sex (f/m) | 22/21  51.1%/48.9% | 37/40  48.0%/52.0% | 15/17  46.9%/53.1% | 0.92 |
| smoker (n/y) | 29/14  67.4%/32.6% | 45/32  58.4%/41.6% | 14/18  43.8%/56.2% | 0.12 |
| disease type (AB/COPD) | 12/31  27.9%/72.1% | 12/65  15.6%/84.4% | 5/27  15.6%/84.4% | 0.22 |
| level of education (high school graduate or higher/no high school diploma) | 10/32  23.8%/76.2% | 18/59  23.4%/76.6% | 5/27  15.6%/84.4% | 0.63 |
| employment (n/y) | 36/6  85.7%/14.3% | 70/7  90.9%/9.1% | 26/6  81.3%/18.7% | 0.35 |
| family status (in relationship/single) | 25/17  59.5%/40.5% | 45/35  58.5%/41.5% | 17/15  53.1%/46.9% | 0.84 |
| number of smokers among close acquaintance (most nonsmoker/most smoker or equal number of smokers) | 32/10  76.2%/23.8% | 54/23  70.1%/29.9% | 26/5  83.9%/16.1% | 0.32 |
